# The insula represents a key neurobiological pain hub in psoriatic arthritis

**DOI:** 10.1186/s13075-025-03526-7

**Published:** 2025-03-31

**Authors:** Flavia Sunzini, Kristian Stefanov, Salim Al-Wasity, Chelsea Kaplan, Andrew Schrepf, Noah Waller, Steven Harte, Richard Harris, Daniel J. Clauw, John McLean, Stefan Siebert, Carl S. Goodyear, Gordon D. Waiter, Neil Basu

**Affiliations:** 1 School of Infection & Immunity, University of Glasgow, Glasgow, Scotland, UK; 2 Chronic Pain and Fatigue Research Centre, Department of Anesthesiology, University of Michigan Medical School, Ann Arbor, MI USA; 3 Susan Samueli Integrative Health Institute, School of Medicine, University of California at Irvine, Irvine, CA USA; 4 Department of Anesthesiology and Perioperative Care, School of Medicine, University of California at Irvine, Irvine, CA USA; 5 Department of Clinical Physics and Bioengineering, NHS Greater Glasgow and Clyde, Glasgow, UK; 6 Aberdeen Biomedical Imaging Centre, University of Aberdeen, Aberdeen, UK

**Keywords:** Chronic pain, Psoriatic arthritis, Neuroimaging, Fibromyalgia, Insula

## Abstract

**Background:**

Pain remains a principal complaint for people with psoriatic arthritis (PsA), despite successful mitigation of inflammation. This situation alludes to the co-existence of distinct pain mechanisms. Nociceptive and nociplastic mechanisms are clinically challenging to distinguish. Advances in brain functional magnetic resonance imaging (fMRI) have successfully characterised distinct pain mechanisms across several disorders, in particular implicating the insula. This is the first study to characterise neurobiological markers of pain mechanisms in PsA employing fMRI.

**Methods:**

PsA participants underwent a 6-minutes resting-state fMRI brain scan, and questionnaire assessments of nociplastic pain (2011 ACR fibromyalgia criteria) and body pain, assessed using the Numeric Rating Scale (NRS, 0-100). Functional connectivity between insula seeds (anterior, mid, posterior), and the whole brain was correlated with the above pain outcomes correcting for age and sex, and false discovery rate (FDR) for multiple comparisons.

**Results:**

A total of 46 participants were included (age 49 ± 11.2; 52% female; FM score 12.5 ± 5.7; overall pain 34.8 ± 23.5). PsA participants with higher fibromyalgia scores displayed increased connectivity between: (1) right anterior insula to DMN (*P* < 0.05), (2) right mid and left posterior insula to parahippocampal gyri (*P* < 0.01 FDR); and (3) right mid insula to left frontal pole (*P* = 0.001 FDR). Overall pain was correlated with connectivity of left posterior insula to classical nociceptive regions, including thalamus (*P* = 0.01 FDR) and brainstem (*P* = 0.002 FDR).

**Conclusion:**

For the first time, we demonstrate objectively that nociceptive and nociplastic pain mechanisms co-exist in PsA. PsA pain cannot be assumed to be only nociceptive in origin and screening for nociplastic pain in the future will inform supplementary analgesic approaches.

**Supplementary Information:**

The online version contains supplementary material available at 10.1186/s13075-025-03526-7.

## Introduction

Psoriatic arthritis (PsA) is a chronic immune-mediated disease characterised by painful articular and peri-articular musculoskeletal inflammation. Pain represents a major burden in this population, impacting quality of life and daily function [1]. The currently available immunotherapies offer comprehensive control of inflammation for most patients and subsequent analgesic effects; however, approximately 1/3 of patients continue to report significant pain despite successful immune-therapeutic intervention. This situation suggests the involvement of multiple pain mechanisms in patients with PsA [2, 3].

The International Association for the Study of Pain has classified pain mechanisms into three broad groups: nociceptive, neuropathic, and nociplastic [4]. While nociceptive pain arises from tissue damage and inflammation and neuropathic pain is due to lesions or diseases affecting the somatosensory system, nociplastic pain involves altered nociception without clear tissue damage or somatosensory lesions. Systemic inflammation, which subserves PsA, peripherally activates nociceptors that trigger the transmission of neural signals to the thalamus and sensorimotor cortices in the brain via spinothalamic pathways. There is additional evidence that neural signals associated with systemic inflammation are processed by the insula [5]. The insula (IC) is complex and is divided into posterior and anterior subregions, which are distinct in terms of cytoarchitecture and function (posterior-peripheral stimuli intensity; anterior-salience, affective and emotion integration) [6, 7]. To date, a single case report study has demonstrated correlations between reported pain and left anterior IC activity induced by evoked pain in an individual with PsA [8]. The evidence in rheumatoid arthritis (RA) patients is more extensive; the increased activation of the insula to inflamed joint stimulation suggests a failure of top-down regulation [9], whereas reduced insula activation is correlated with rapid pain relief following TNF-alpha inhibition prior to any detectable anti-inflammatory effects [10]. In addition to encoding for nociceptive pain intensity [11], the insula is commonly implicated in fibromyalgia (FM), the prototypical nociplastic pain disorder. Functional magnetic resonance imaging (fMRI) studies have consistently demonstrated altered insular activity in FM patients [7, 12–14]. Enhanced functional connectivity (synchronous activity across brain regions) of the insula with the default mode network (DMN) is also consistently observed in FM [15]. The DMN refers to a set of brain regions with synchronised activity at rest that are active during self-reflection and includes areas such as the posterior cingulate cortex, parahippocampal gyri, and medial prefrontal cortex (mPFC). Functional connectivity between the DMN and the posterior insula has also been shown to correlate with co-existing FM severity in RA [16], suggesting that DMN-insula connectivity may be a consistent neural marker of nociplastic pain across different conditions, including inflammatory arthritis.

In clinical settings, FM is also more frequently diagnosed in individuals with PsA (9.3–38.3%) [17–21] than in the general population (approximately 1.78%) [22]. Furthermore, distinguishing between inflammation-related pain and nociplastic pain is clinically challenging, as the widespread and difficult-to-localise pain associated with entheseal inflammation, which characterises PsA but not RA, often resembles the diffuse pain and tenderness observed in FM and the subsequent inflation of disease activity indices, which all include pain, potentially leading to inappropriate treatment escalation [23].

Although active PsA is primarily characterised by nociceptive pain driven by inflammation, clinical observation alludes to the co-existence of nociplastic pain mechanisms. However, to date, no objective neurobiological evidence has been provided to substantiate this observation in PsA. This study represents the first attempt to neurobiologically characterise pain in PsA. It focuses on the insula given its well-established role in both nociceptive and nociplastic pain mechanisms in other chronic pain populations [1–4]. We specifically hypothesise that PsA patients with the higher degrees of nociplastic pain will present heightened DMN–insula functional connectivity, consistent with findings observed in FM and RA. Second, we examine the role of the insula in overall body pain, as measured by rsting-state functional connectivity fMRI. Through this analysis, we seek to determine whether altered insula function in PsA underpins widespread pain perception extending beyond localized inflammation, highlighting the interplay and potential divergence between nociplastic and nociceptive pain mechanisms in PsA.

## Methods

### Study design and participants

Participants with active PsA were recruited from specialist rheumatology clinics in a single-centre cross-sectional observational study from June 2019 to October 2021. Participants were considered eligible if they (1) were aged over 18 years old at the time of consent; (2) fulfilled the CASPAR criteria for PsA [24]; (3) had active joint disease according to the judgement of the rheumatologist and were being started on a new treatment with either a biologic or a DMARD(s); (4) were right hand dominant to avoid heterogeneity in the MRI analysis; and (5) had no contraindications to MRI (e.g., severe claustrophobia).

Before starting the newly prescribed biologic/csDMARD, all consenting subjects attended a research visit, which included a standardised clinical evaluation and an hour-long fMRI brain scan.

The clinical evaluation included our outcomes of interest: (1) the 2011 ACR FM criteria [25], a proposed proxy measure of nociplastic pain previously validated in a RA cohort with fMRI [16]. Participants were classified as having FM if they met the 2011 ACR FM criteria, while total FM scores (0–31) were used as a continuous variable to express the degree of nociplastic pain (FMness), independent of the FM classification [15]. (2) Overall body pain intensity was collected before the MRI scan on a numeric rating scale (NRS, 0-100). In addition, demographic data (age, sex, BMI measured at the time of the visit), disease history based on medical records (onset, previous exposure to Disease-Modifying Antirheumatic Drugs (DMARDs)—a class of medications designed to slow the progression of inflammatory arthritis and prevent joint damage—current medications), and PsA disease activity data (swollen and tender joint counts of 66 and 68, respectively, CRP, global VAS assessment, Disease Activity in Psoriatic Arthritis (DAPSA), Leeds Enthesitis Index (LEI), and Bath Ankylosing Spondylitis Disease Activity Index (BASDAI)) were collected at the time of the visit.

### MRI data acquisition and preprocessing

Participants undertook scans on a 3 Tesla Siemens PRISMA (Siemens, Erlangen, Germany) in Glasgow (UK) using the body transmit and 32 channel phased-array receive-only head coil. These included a T1-weighted fast-field echo 3D structural image (TR = 2500 ms, TE = 2.88 ms, inversion time (TI) = 1070 ms, flip angle = 8°, FOV = 256 mm, with 176 slices, 1 mm iso-voxel) and functional images obtained via a T2*-weighted multiband EPI sequence (TR = 800 ms, TE = 30 ms, flip angle = 52°, FOV = 216 mm, acceleration factor = 6, 60 slices, 440 volumes at 2.4 mm iso-voxel). A 6-minute resting-state fMRI scan was performed for the analysis, during which individuals were asked to lie supine in the scanner and keep their eyes open, focusing on a fixation cross without engaging in any specific task.

Preprocessing and later analysis of the images were carried out using SPM12 within the functional connectivity CONN toolbox v19 [26], running in MATLAB R2019b. Preprocessing included the default MNI pipeline by the functional connectivity toolbox CONN: realignment, slice-timing correction, ART-based motion outlier detection, coregistration, functional and structural segmentation, Montreal Neurological Institute (MNI) template normalisation and 8-mm smoothing (convolution with an 8 mm full-width at half maximum Gaussian Kernel). The choice of smoothing kernel was done to both follow the standard pipeline in the CONN toolbox as well as reproduce the pipeline used in the previous study in RA patients. All the scans were visually inspected for artefacts. Individual volumes were omitted from the analysis if they had over two millimetres of motion and a global BOLD signal of over nine standard deviations. A patient was considered for exclusion from analysis if more than 20% of their functional volume was omitted (88 volumes). Signals from white matter and cerebrospinal fluid were extracted via the CompCor procedure, and motion parameters were entered into the analysis as covariates of no interest via ordinary least squares regression. A bandpass filter (frequency window: 0.008–0.09 Hz) was applied to remove linear drifts and high-frequency noise from the data.

### Functional connectivity analysis pipelines

**Fig. 1:**
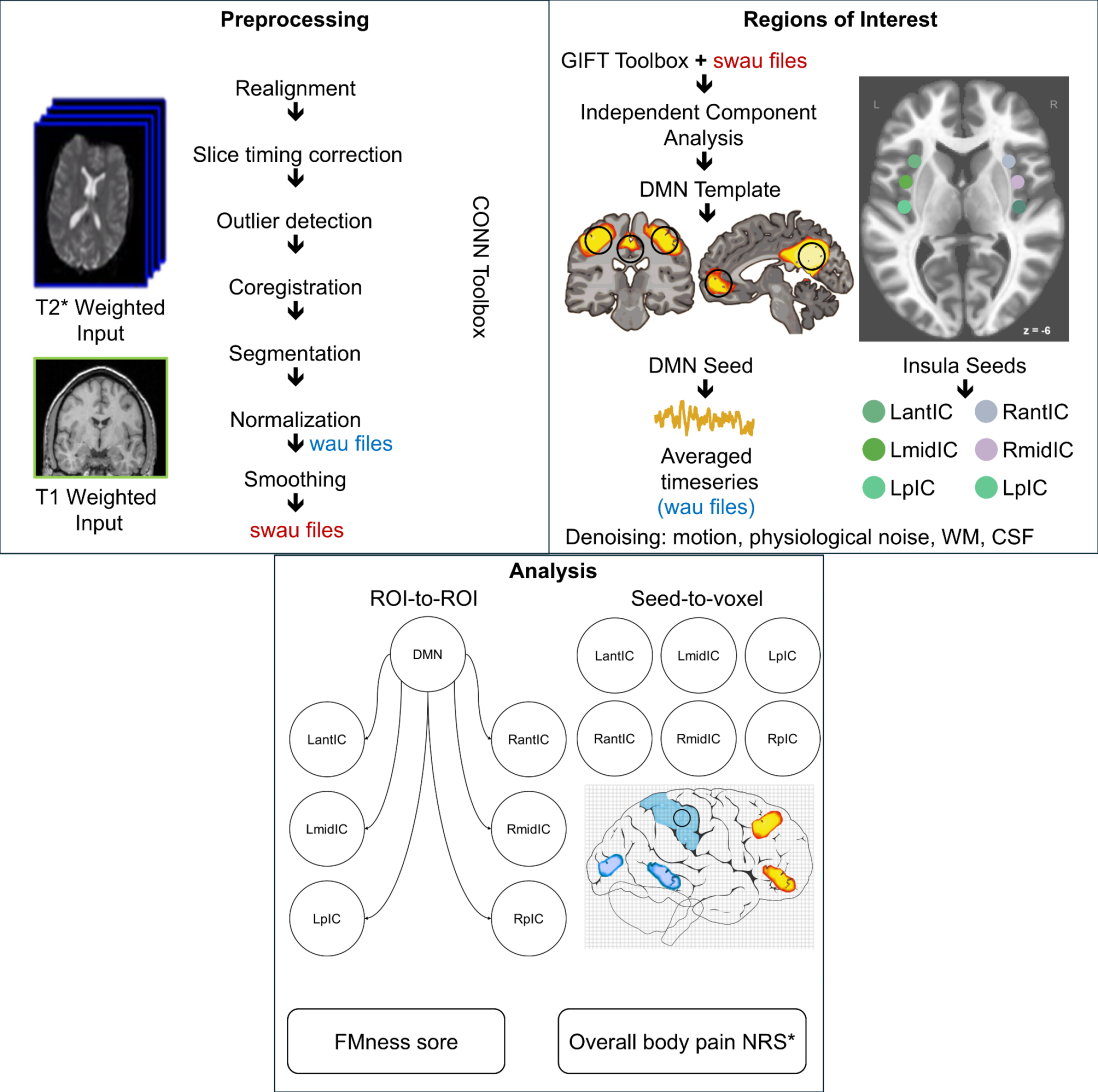
Functional connectivity pipelines. Preprocessing panel displays the default MNI pipeline in the CONN toolbox that requires both structural (T1 weighted) and functional (T2* weighted) MRI images. The preprocessed smoothed (swau) images were then used for. independent component analysis (ICA), which along with a default mode network (DMN) template identified the DMN. Six insula subregions were also extracted as spheres of 6 mm surrounding a peak voxel. Montreal Neurological Institute (MNI) coordinates for each voxel include left anterior insula (LantIC): x =– 32, y = 16, z = 6; left mid insula (LmidIC): x =– 38, y = 2, z = 8; left posterior insula (LpIC): x =– 39, y =– 15, z = 1; right anterior insula (RantIC): x = 32, y = 16, z = 6; right mid insula (RmidIC): x = 38, y = 2, z = 8; right posterior insula (RpIC): x = 39, y =– 15, z = 8. The timeseries of the DMN and insula subregions were extracted from the preprocessed unsmoothed (wau) images and denoised for motion, physiological noise (band-pass filtering) and white matter (WM) and cerebrospinal fluid (CSF) signals. Functional connectivity between the DMN and the six insula regions were used in a region-of-interest (ROI) analysis to look for associates with FMness score. A seed to voxel analyses was also run with all seven regions for FMness score but only the posterior insula regions for the overall body pain NRS. Adapted from Scheinost et al. (2017), while the figure was partly generated using Servier Medical Art, licensed under a Creative Commons Attribution 3.0 (unported license) and edited using Inkscape (2020).

The classical approach to estimating functional connectivity (FC) is to calculate Pearson correlation coefficients between two time series of BOLD signal changes measured throughout the sequence. The time series are the low-frequency changes in BOLD signals from a source (seed) and a target location. The seed is typically a brain region-of-interest (ROI), which is a set of voxels that encompass an anatomically distinct brain region. The target can be the time-course of a single voxel (seed-to-voxel) or a time-course averaged across all the voxels of another ROI (ROI-to-ROI). To evaluate whether PsA patients with nociplastic pain present with heightened DMN-insula connectivity, we computed the ROI-to-ROI functional connectivity between the DMN and different regions of the insula as well as seed-to-voxel connectivity using insula subregions as seeds (Fig. 1). To characterise the role of the insula in overall body pain, we computed similar seed-to-voxel connectivity of the whole brain with posterior regions of the insula and correlated this connectivity with overall body pain intensity scores. The posterior insula was chosen because of its established role in pain intensity [11].

Group independent component analysis (Fig. 1) was performed to identify the DMN using the Group ICA of the fMRI Toolbox (GIFT) toolbox [27]. Employing the pre-processed functional data, component estimates were validated via the Infomax ICA algorithm 20 times in ICASSO software. Similar to analyses in rheumatoid arthritis [16, 28, 29], 40 components were used to identify cortical and subcortical components that correspond to brain networks. Subject-specific spatial maps and time courses were estimated using the GICA back reconstruction method. The DMN was confirmed by spatial correlation between component maps and a published template map from Beckmann et al. [30], who looked at intrinsic connectivity that also strongly overlaps with task-based connectivity-derived networks [31]. The spatial mask of the mean component map for the DMN was created via the MarsBaR toolbox for seed-based functional connectivity analyses. Seed-based connectivity was also estimated from distinct insula subregions. The subregions were defined based on previous findings in patients with FM, whose connectivity was associated with pain thresholds [7], originally defined by Taylor et al. [32]. The regions included were the anterior, middle, and posterior insula bilaterally. The seed regions were created as spheres (6-mm diameter) using the MarsBaR toolbox. In addition to seed-to-voxel analysis, these regions were also used in an ROI-to-ROI analysis with the DMN (Fig. 1**)**.

The abovementioned seed-to-voxel and ROI-to-ROI connectivity were implemented in general linear models (GLMs) in the CONN toolbox to identify associations of nociplastic pain (FMness) and overall body pain while controlling for age and sex as covariates of no interest. The resulting seed-to-voxel maps were thresholded at a whole-brain *p* < 0.001, uncorrected voxel threshold and *p* < 0.05 false discovery rate (FDR) cluster corrected for multiple comparisons. The ROI-to-ROI connectivity analyses were thresholded at *p* < 0.05.

## Results

### Population characteristics and pain outcomes

Among the 50 subjects recruited, MRI data were not available for four participants (one with nonremovable piercing, two with claustrophobia, and one with poor brain coverage). None of the subjects exceeded the motion or global BOLD signal criteria for exclusion (mean motion (SD) = 0.151 (0.063), mean excluded volume (SD) = 2.696 [6]). The baseline characteristics of the recruited population are described in Table 1 and did not differ significantly from those of the excluded patients (Table S1). The participants included in the analysis were middle-aged (mean age 49 ± 11.2 years), with a mild prevalence of females (52%), and the PsA disease duration was variable on the basis of medical records (mean 6 years ± 5.8 years). In the study cohort, the participants predominantly fell within the overweight to obese range (mean BMI 29 ± 4.5). Most participants (90%) had concomitant psoriatic skin involvement and additional comorbidities: diabetes (*n* = 4), gout (*n* = 1), hypertension (*n* = 5), and scleritis/uveitis (*n* = 4). Five participants were prescribed their first immunosuppressive drug, 23 were biologic-naïve, and 10 were switching from their first biologic due to treatment failure (9 anti-TNF agents and 1 anti-IL-17 A agent), whereas 4 subjects had not responded to at least the fourth line of biologics. Overall, the clinical parameters of disease activity were increased (Table 1), reflecting high disease activity per the inclusion criteria. Notably, all the participants presented with at least one swollen joint. The CRP normal range defined in local NHS laboratories is 1 mg/dl; most participants had CRP levels within the normal range (mean 1 ± 2.5).

**Table 1:** Baseline clinical characteristics of participants included in the analysis

Clinical features (*n* = 46)	
Age (mean ± SD)	49 ± 11.2
Disease duration (years, mean ± SD)	6.4 ± 5.8
Sex (male/female)	22/24
BMI (mean ± SD)	29 ± 4.5
FM criteria fulfilled (%)	43.5%
FM total score (mean ± SD)	12.5 ± 5.7
Current overall body pain NRS 0-100 (mean ± SD)	34.8 ± 23.5
Number of previous DMARDs (including biologics) • 0–1 • 2–4 • > 4	17 17 12
TJC 66 (mean ± SD)	21 ± 14
SJC 68 (mean ± SD)	7 ± 4.3
CRP (mg/dL, mean ± SD)	1 ± 2.5
Patient gVAS (mean ± SD)	59 ± 22
DAPSA (mean ± SD)	40.8 ± 19
LEI score (mean ± SD)	2.7 ± 2
BASDAI (mean ± SD)	6.2 ± 1.8

BASDAI– Bath Ankylosing Spondylitis Disease Activity Index; BMI– Body Mass Index; DAPSA– Disease Activity in PSoriatic Arthritis; FM– Fibromyalgia; gVAS– Global Disease Activity; LEI– Leed Enthesitis Index; NRS– Numeric Rating Scale; SD– Standard Deviation; SJC– Swollen Joints Count; TJC– Tender Joints Count

Current overall body pain was used as a measure of nociceptive and mixed pain in this cohort of individuals with active PsA. Pain was assessed using a NRS scale (0–100) that describes current overall body pain in each participant before they entered the MRI scanner (mean 34.8 ± 23.5). The degree of nociplastic pain in the recruited subjects was determined with the total score of the 2011 ACR FM criteria (FigureS1), defined as FMness, whether the participants fulfilled the criteria or not. Nonetheless, 20 subjects met the ACR classification criteria (43.5%), suggesting a high prevalence of comorbid FM in the recruited population, which is slightly higher than expected on the basis of the current literature [17–21]. Clinical differences between subjects meeting or not meeting the 2011 ACR FM criteria are available in Table 2. The current overall level of body pain was slightly greater in the participants with FM (40 ± 26.3 versus 30.8 ± 20.7); however, the difference was not significant. Subjects with comorbid FM had significantly higher TJC and patient gVAS scores (*p* = 0.004 and *p* = 0.007, respectively), likely contributing to the significantly higher DAPSA scores than the participants without FM (*p* = 0.0010). Both LEI and BASDAI scores were also significantly greater in the FM group (p values of 0.04 and 0.005, respectively). The number of previous antirheumatic drugs was also significantly greater in the participants with FM (*p* = 0.003), despite similar PsA disease durations.

**Table 2:** Differences in clinical characteristics between study participants with and without fibromyalgia as a comorbidity

Clinical features	With Fibromyalgia (*n* = 20)	Without Fibromyalgia (*n* = 26)	*P*-value	Effect Size (Cohen’s d)
Age (mean ± SD)	50 ± 9.7	47.8 ± 12.4	ns	0,21
Disease duration (years, mean ± SD)	5.2 ± 4.8	7 ± 6.5	ns	0.31
Sex (male/female)	7/13	15/11	ns	0.46
BMI (mean ± SD)	30.3 ± 4.1	28.8 ± 4.8	ns	0.34
FM criteria fulfilled (%)	100%	0%		
FM total score (mean ± SD)	17.8 ± 3.2	8.4 ± 3.4	< 0.0001	2.89
Current overall body pain NRS 0-100 (mean ± SD)	40 ± 26.3	30.8 ± 20.7	ns	0.4
Number of previous DMARDs (including biologics) 0–1 2–4 > 4	4 4 12	10 13 3	0.002	1.23
TJC 66 (mean ± SD)	27.2 ± 14.1	16 ± 11.8	0.004	0.86
SJC 68 (mean ± SD)	8.2 ± 5.2	5.9 ± 3.3	ns	0.54
CRP (mg/dL, mean ± SD)	1.7 ± 3.7	0.5 ± 0.5	ns	0.48
Patient gVAS (mean ± SD)	50.9 ± 20.3	33 ± 13.5	0.007	1.07
DAPSA (mean ± SD)	67.8 ± 17.9	52 ± 23	0.001	0.75
LEI score (mean ± SD)	3.4 ± 2	2.2 ± 1.8	0.034	0.66
BASDAI (mean ± SD)	7 ± 1.5	5.5 ± 1.8	0.005	0.87

BASDAI– Bath Ankylosing Spondylitis Disease Activity Index; BMI– Body Mass Index; DAPSA– Disease Activity in PSoriatic Arthritis; FM– Fibromyalgia; gVAS– Global Disease Activity; LEI– Leed Enthesitis Index; NRS– Numeric Rating Scale; SD– Standard Deviation; SJC– Swollen Joints Count; TJC– Tender Joints Count. Significant differences were determined via unpaired t tests and chi-square tests. Effect Size (Cohen’s d) threshold for interpretation: d < 0.2 - small effect; d = 0.5 - medium effect; d = 0.8 - large effect

### DMN-to-right anterior insula functional connectivity is altered in patients with PsA with a high degree of nociplastic pain

**Fig. 2:**
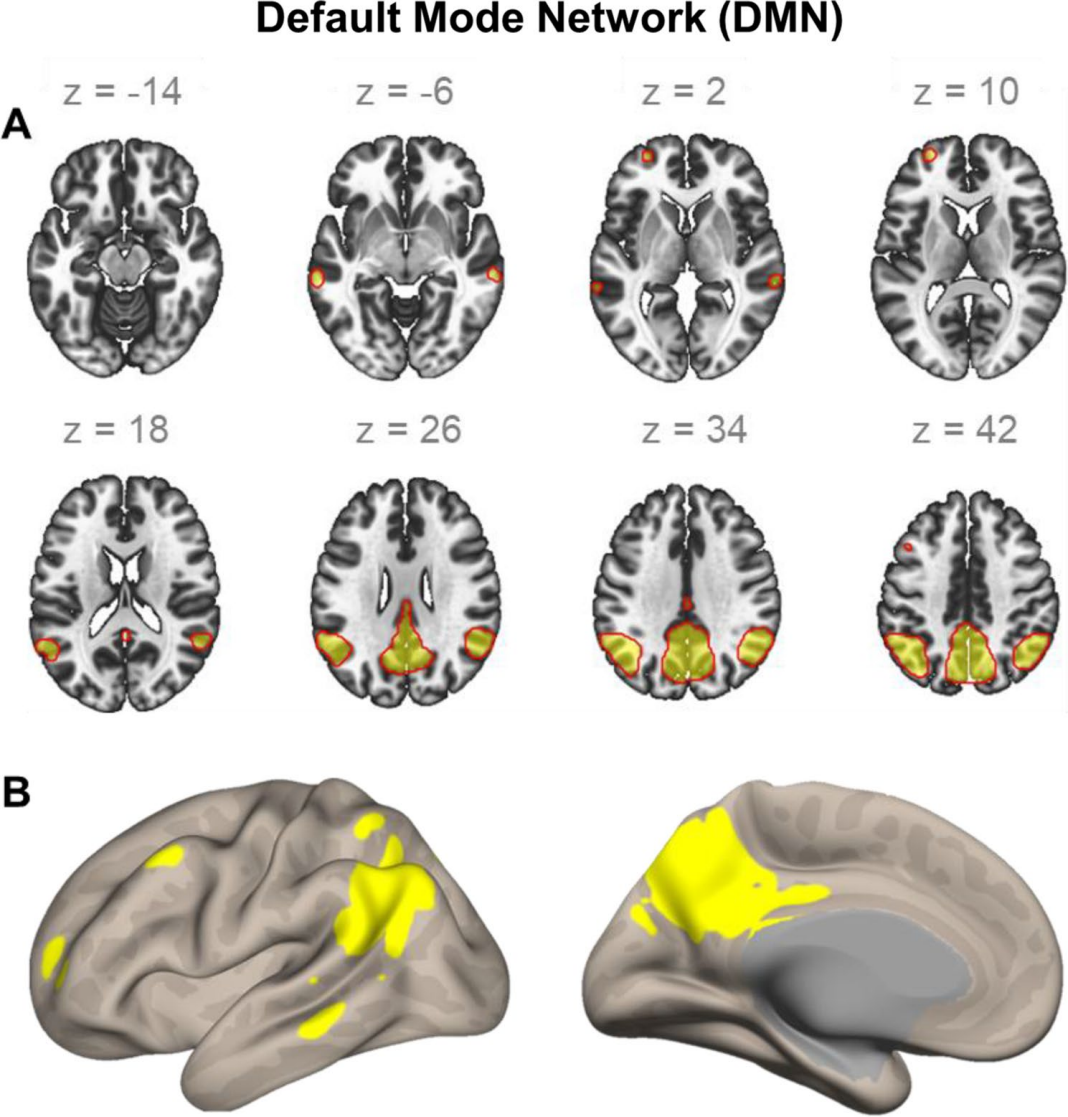
The default mode network (DMN) in patients with psoriatic arthritis. Visualisation of the DMN in volumetric space (panel A) in an axial view at different slices (z:– 14,– 6, 2, 10, 18, 26, 34, 42). Panel B displays the same network in surface space in left lateral and medial view. Both visualisations were created in the CONN toolbox (Nieto-Castanon, 2020)

**Fig. 3:**
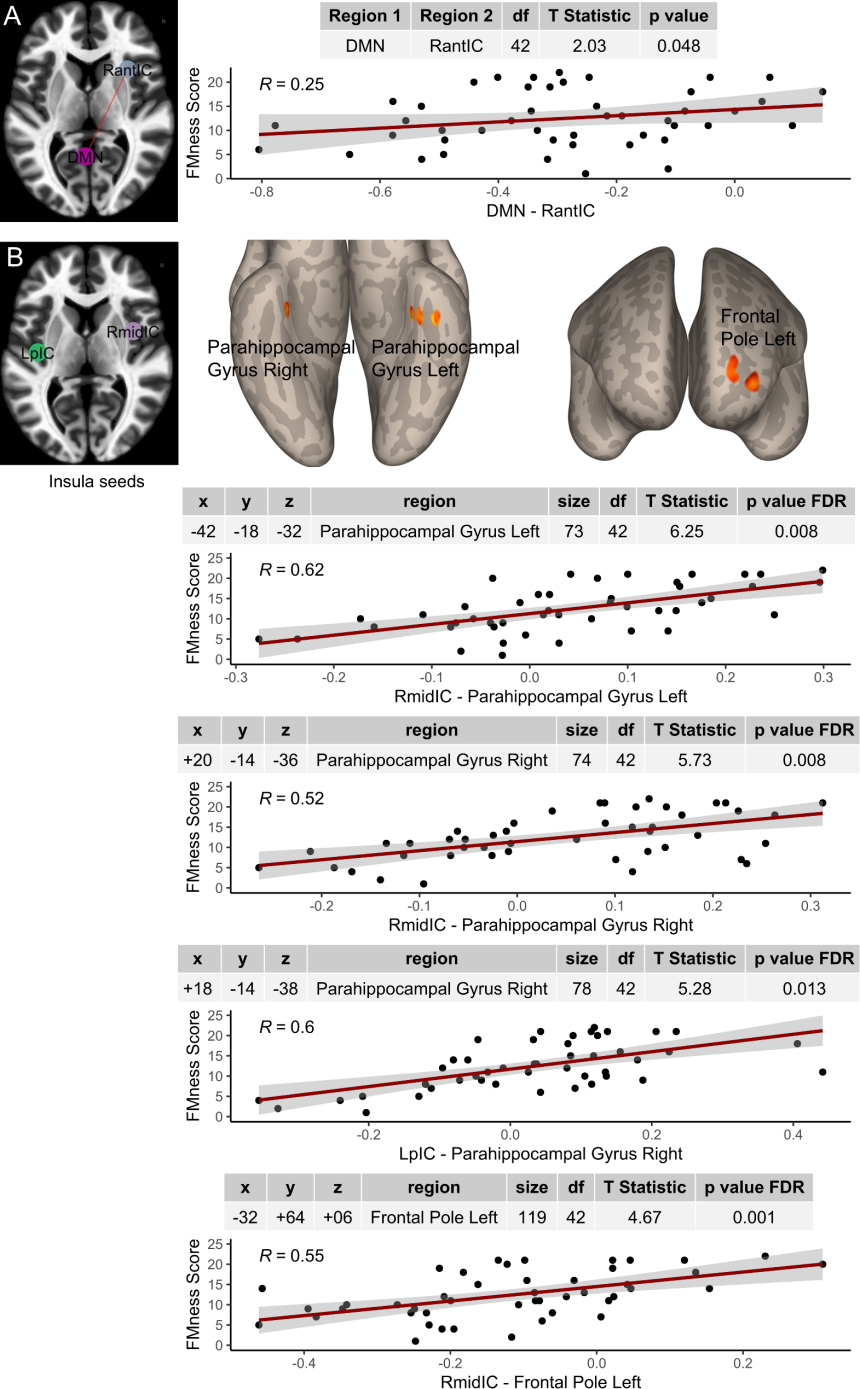
DMN-to-insula connectivity is associated with nociplastic pain. **Panel A** The left image visualises the seed of the right anterior insular cortex (RantIC) and the default mode network (DMN), which is pictured mostly in the posterior cingulate cortex for visualisation purposes. The images on the right visualise a scatterplot (95% confidence intervals) of the functional connectivity (Fisher z-transformed r values) between the DMN and RantIC and nociplastic pain scores and their Pearson correlation coefficient (R). The table above the plot also displays the degrees of freedom (df), test statistic (t statistic) and p value of the general linear model between functional connectivity and nociplastic pain while controlling for age and sex. **Panel B** The top images visualise the right mid-insular cortex (RmidIC) and left posterior insular cortex (LpIC) seeds and the clusters of voxels within the left and right parahippocampal gyri as well as the left frontal pole, with which functional connectivity was associated with nociplastic pain while controlling for age and sex in a seed‒to-voxel analysis. The images below display the scatterplots, statistical tables, peak voxel coordinates in MNI space (x, y, z) and cluster size, with the p values after false discovery rate (FDR) correction for multiple comparisons of the general linear models.

The ROI-to-ROI analysis investigated the associations between the total FMness scores and the functional connectivity between the 6 insula seeds and the DMN identified via ICA (Fig. 2). The functional connectivity between the DMN and the right anterior insula was positively associated with the degree of nociplastic pain in the study participants with PsA (Fig. 3A). Specifically, the connectivity values extend across the negative Fisher Z-scores, seemingly reflecting different degrees of anti-correlation. Greater anticorrelation was observed in participants with lower FMness scores, whereas subjects with higher FMness presented a reduced anticorrelation between the DMN and insula. No other significant correlations emerged from the ROI-to-ROI analyses when the selected insula seeds were used (Table S2).

An additional seed-to-voxel analysis supported the investigation of the functional connectivity of the 6 insula seeds with the whole brain. Nociplastic pain measures (i.e., FMness scores) were positively correlated with connectivity between the right middle insula and left posterior insula, with clusters in the left and right parahippocampal gyri, as well as in the frontal pole, which covers the mPFC. Both brain regions, the parahippocampal gyri and mPFC, are part of the DMN, suggesting that altered insula functional connectivity to specific regions of the DMN is associated with the degree of nociplastic pain in this PsA cohort (Fig. 3B).

### Overall body pain is associated with altered connectivity of the posterior insula with classical nociceptive brain regions

**Fig. 4:**
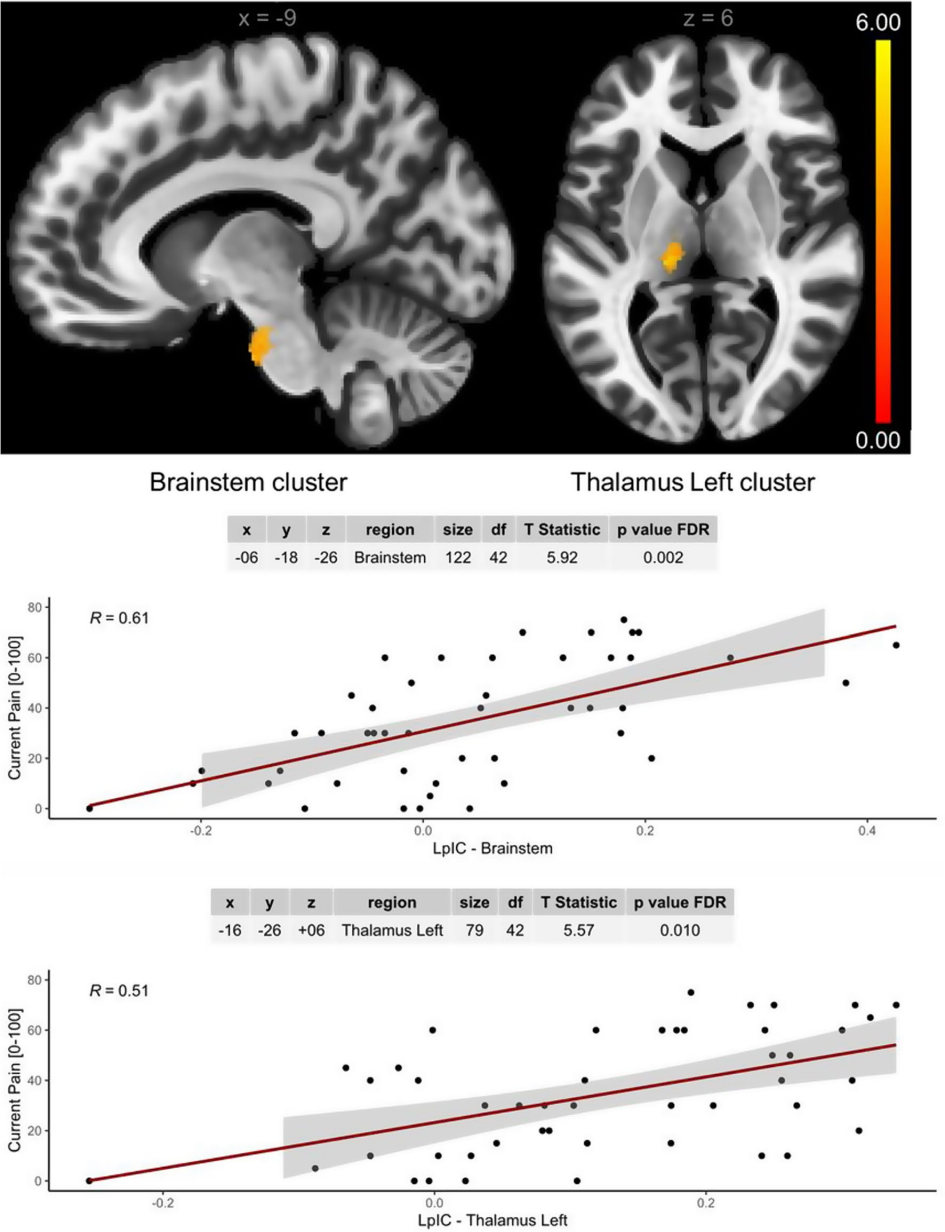
Left posterior insula connectivity to the brainstem and thalamus is associated with current pain. The panels above display the clusters in the brainstem and left thalamus whose connectivity with the left posterior insular cortex (LpIC) was associated with current overall body pain while controlling for age and sex. The panels below display the scatterplots (95% confidence intervals) with Pearson correlation coefficients (R) between the functional connectivity (Fisher z transformed r values) of the seeds and clusters with current overall body pain. The tables describe the peak voxel coordinates in MNI space (x, y, z), the cluster size, the degrees of freedom (df), the T statistics, and the p values after false discovery rate (FDR) correction for multiple comparisons of the general linear models (GLMs)

Seed-to-voxel analysis revealed positive associations between current overall body pain and functional connectivity of the left posterior insula to the brainstem (the pons) and the ipsilateral thalamus (Fig. 4). Similar correlations with overall body pain were also found with left posterior insula connectivity to the middle temporal gyrus and to the right insula, as well as between the right posterior insula and the cerebellum (Table S2).

## Discussion

This is the first study to directly investigate the neurobiological markers of pain in PsA patients. In line with other chronic pain conditions, the insula appears to be a key brain region in PsA pain processes. Specifically, increased functional connectivity with the DMN was significant among patients with clinical features of FM, thus providing objective evidence that nociplastic pain mechanisms are present in PsA.

We have supported our primary hypothesis that a neurobiological marker of nociplastic pain, DMN-insula altered functional connectivity, is observed among PsA patients with clinical features of FM, as we previously observed in RA [16]. Notably, we found that PsA patients with higher FMness scores presented weaker anticorrelations between the DMN and the right anterior insula. In healthy subjects, the DMN is inversely correlated with the salience network, a major component of which is the anterior insula [33]. In chronic pain, this anti-correlation is lost [34], as we observed in our study among PsA participants with high FMness scores. However, it is important to note that the significance of this finding (*p* = 0.048) is marginal and should therefore be interpreted with caution. While this result aligns with previous studies in FM and other chronic pain conditions, further investigation is needed to establish the robustness of this observation and its broader implications. Nonetheless, the whole-brain approach revealed FM-related functional connectivity between the bilateral mid-insula and the parahippocampal gyri, as well as between the right mid-insula and the frontal pole. Both the parahippocampal gyrus and the frontal pole are part of the DMN; thus, these findings further validate the presence of altered DMN–insula connectivity in PsA. Interestingly, parahippocampal regions have recently emerged as key areas involved in nociplastic pain. Previous studies have demonstrated increased parahippocampal activation in FM at rest and after task-evoked scans [35–37]. Moreover, both the parahippocampal gyrus and the anterior insula are part of the descending pain inhibitory pathway, which has been shown to be diminished in primary FM [38, 39].

In addition to FMness scores, overall body pain intensity was explored [40]. While acknowledging that a pain NRS cannot differentiate between different pain mechanisms, in the context of patients with active inflammatory arthritis, it is reasonable to assume a major contribution of nociceptive mechanisms. In this cohort, current overall body pain was not significantly greater in the participants fulfilling the 2011 ACR FM criteria (Table 2). This finding suggests that pain mechanisms are not easily distinguished using the current overall pain only, at least when high nociceptive input is present (i.e., inflammation). Moreover, we found that current overall body pain intensity is associated with left posterior insula connectivity to the pons of the brainstem (spinothalamic tract) and the left thalamus, brain regions classically implicated in somatosensory nociceptive pathways [41], suggesting a nociceptive contribution to PsA pain.

The findings also highlight the distinct functional implications of different insular regions. The right anterior insula, a key region of the salience network, is heavily involved in integrating emotional and attentional aspects of pain and is particularly implicated in the cognitive evaluation of pain intensity and unpleasantness. The loss of anti-correlation between the right anterior insula and the DMN in PsA with high FMness scores may reflect the sustained engagement of the salience network in the presence of chronic pain, disrupting the balance between pain-related salience and the internally directed processes mediated by the DMN [6, 12]. Conversely, the left posterior insula, which showed functional connectivity with brainstem regions such as the pons and thalamus in this study, is classically associated with the somatosensory processing of nociceptive inputs. Therefore, the posterior insula functional connectivity likely represents the contribution of peripheral nociceptive pathways to overall pain in PsA, particularly in patients with active inflammation.

Several factors may influence our conclusions. First, the study’s generalisability is limited because recruitment is restricted to patients with active disease. Consequently, such selection would enlist patients with a greater predominance of nociceptive pain. Despite this dilution, the observed significant DMN-insula observations emphasise the high relevance of nociplastic pain in PsA. Second, despite being one of the largest MRI brain studies on PsA pain reported in the rheumatology literature to date, some potential confounding effects could not be controlled due to limitations in sample size. For example, cognitive function could be an important factor, as the parahippocampal gyri is not only a part of the DMN but also associated with learning, memory, and internal body mapping. However, we were powered to control for age and sex. The latter is especially important in recognition of the apparent sex differences commonly observed in the pain neurobiology literature [42, 43]. The cross-sectional design of the study also meant that no causal inference could be made on the association between DMN-insula connectivity and FM pain. Finally, we opted not to use other methodological strategies like fmriprep preprocessing pipeline and surface-based analysis that improves on BOLD signal localization [44]. Since our hypotheses were driven by the previous findings in primary fibromyalgia and RA, we opted to reproduce those preprocessing and analysis pipelines, which are still a default option in the CONN toolbox and comparable to other methodological strategies [45].

To note, is the higher prevalence of FM in our cohort compared to the existing literature, which has a potential impact on the study findings and interpretations. This is likely to reflect a combination of factors. The recruitment criteria, which selected for PsA patients with active disease requiring treatment escalation, may have inadvertently enriched the cohort with individuals experiencing heightened nociplastic pain symptomatology. Additionally, referral and participation bias could have played a role, as individuals with chronic and severe pain, including FM-like symptoms, may have been more likely to be referred or motivated to join a study focusing on pain mechanisms. While no specific data are available to confirm regional variations, the local Scottish community may also have a higher prevalence of FM compared to other populations. However, the small sample size and single-centre design limit the generalizability of our findings, and future multicentre studies are needed to validate these observations. Moreover, case-control studies would aid in the comparisons of PsA patients with other cohorts, e.g., healthy controls, FM patients, and RA patients. Future longitudinal studies should examine these biomarkers in response to therapies or directly target them through non-invasive brain modulation techniques.

This study highlights the co-existence of distinct pain mechanisms in PsA, which require distinct therapeutic approaches. While immune-modulating therapeutics are conventionally considered first-line therapies for PsA pain, they are designed to target the contribution of inflammation-mediated nociceptive pain. In contrast, nociplastic pain mechanisms are more optimally attenuated by non-pharmacological strategies, such as exercise and cognitive behavioural programs, and centrally active compounds, such as amitriptyline [45]. The highlighted MRI biomarkers may form the basis of future tools to enable more precise pain stratification, but in the interim, these data should encourage clinicians to consider the balance of these mechanisms within their individual patients by employing clinical phenotyping, such as the ACR FM scale.

## Conclusions

The findings of this study suggest a key role of the insula in pain perception in PsA patients. The degree of nociplastic pain in PsA is associated with altered connectivity between mid-anterior insula regions and the DMN, similar to primary FM; additionally, the posterior insula appears to communicate strongly with brain regions established to be important in nociceptive pain pathways. Bridging the gap between clinical observations and neurobiological evidence of nociplastic pain in PsA is crucial for a comprehensive understanding of the underlying pain mechanisms, ultimately helping the development of tailored pain management strategies to improve the well-being of individuals affected by PsA.

## Electronic supplementary material

Below is the link to the electronic supplementary material.

Supplementary Material 1

## Data Availability

No datasets were generated or analysed during the current study.

## References

[1] Sumpton D, Kelly A, Tunnicliffe DJ, Craig JC, Hassett G, Chessman D, et al. Patients’ perspectives and experience of psoriasis and psoriatic arthritis: A systematic review and thematic synthesis of qualitative studies. Arthritis Care Res. 2020;72(5):711–22. 10.1002/acr.23896 30927508

[2] Rutter-Locher Z, Arumalla N, Norton S, Taams LS, Kirkham BW, Bannister K. A systematic review and meta-analysis of questionnaires to screen for pain sensitisation and neuropathic like pain in inflammatory arthritis. Semin Arthritis Rheum. 2023;61:152207. 37163841 10.1016/j.semarthrit.2023.152207

[3] Gudu T, Gossec L. Quality of life in psoriatic arthritis. Expert Rev Clin Immunol. 2018;14(5):405–17. 29681202 10.1080/1744666X.2018.1468252

[4] Terminology| International Association for the Study of Pain [Internet]. International Association for the Study of Pain (IASP). [cited 2023 Aug 21]. Available from: https://www.iasp-pain.org/resources/terminology/

[5] Kerezoudis P, Howe CL, Wu LJ, Lundstrom BN, Van Gompel JJ. Insula and the immune system: more than Mere Co-existence? Neurosci Bull. 2022;38(10):1271–3. 35763253 10.1007/s12264-022-00911-z PMC9554059

[6] Uddin LQ, Nomi JS, Hébert-Seropian B, Ghaziri J, Boucher O. Structure and function of the human Insula. J Clin Neurophysiol. 2017;34(4):300–6. 28644199 10.1097/WNP.0000000000000377 PMC6032992

[7] Ichesco E, Schmidt-Wilcke T, Bhavsar R, Clauw DJ, Peltier SJ, Kim J, et al. Altered resting state connectivity of the insular cortex in individuals with fibromyalgia. J Pain. 2014;15(8):815–e8261. 24815079 10.1016/j.jpain.2014.04.007 PMC4127388

[8] Baliki M, Katz J, Chialvo DR, Apkarian AV. Single subject pharmacological-MRI (phMRI) study: modulation of brain activity of psoriatic arthritis pain by cyclooxygenase-2 inhibitor. Mol Pain. 2005;1:32. 16266429 10.1186/1744-8069-1-32 PMC1291397

[9] Sandström A, Ellerbrock I, Jensen KB, Martinsen S, Altawil R, Hakeberg P, et al. Altered cerebral pain processing of noxious stimuli from inflamed joints in rheumatoid arthritis: an event-related fMRI study. Brain Behav Immun. 2019;81:272–9. 31228612 10.1016/j.bbi.2019.06.024

[10] Rech J, Hess A, Finzel S, Kreitz S, Sergeeva M, Englbrecht M, et al. Association of brain functional magnetic resonance activity with response to tumor necrosis factor Inhibition in rheumatoid arthritis. Arthritis Rheum. 2013;65(2):325–33. 23238986 10.1002/art.37761

[11] Segerdahl A, Mezue M, Okell T. The dorsal posterior Insula subserves a fundamental role in human pain. Nat Neurosci. 2015;18:499–500. 25751532 10.1038/nn.3969 PMC6783299

[12] Ichesco E, Puiu T, Hampson JP, Kairys AE, Clauw DJ, Harte SE, et al. Altered fMRI resting-state connectivity in individuals with fibromyalgia on acute pain stimulation. Eur J Pain Lond Engl. 2016;20(7):1079–89. 10.1002/ejp.832 26773435

[13] López-Solà M, Woo CW, Pujol J, Deus J, Harrison BJ, Monfort J, et al. Towards a neurophysiological signature for fibromyalgia. Pain. 2017;158(1):34–47. 27583567 10.1097/j.pain.0000000000000707 PMC5161739

[14] Kaplan CM, Schrepf A, Vatansever D, Larkin TE, Mawla I, Ichesco E, et al. Functional and neurochemical disruptions of brain hub topology in chronic pain. Pain. 2019;160(4):973–83. 30763287 10.1097/j.pain.0000000000001480 PMC6424595

[15] Wolfe F, Fibromyalgianess. Arthritis Rheum. 2009;61(6):715–6. 19479689 10.1002/art.24553

[16] Basu N, Kaplan CM, Ichesco E, Larkin T, Harris RE, Murray A, et al. Neurobiologic features of fibromyalgia are also present among rheumatoid arthritis patients. Arthritis Rheumatol. 2018;70(7):1000–7. 29439291 10.1002/art.40451

[17] Kancharla H, Jain S, Mishra S, Acharya N, Grover S, Dogra S, et al. Fibromyalgia influences health-related quality of life and disease activity in psoriatic arthritis. Rheumatol Int. 2022;42(3):511–7. 34251497 10.1007/s00296-021-04925-0

[18] Brikman S, Furer V, Wollman J, Borok S, Matz H, Polachek A, et al. The effect of the presence of fibromyalgia on common clinical disease activity indices in patients with psoriatic arthritis: A Cross-sectional study. J Rheumatol. 2016;43(9):1749–54. 27252430 10.3899/jrheum.151491

[19] Iannone F, Nivuori M, Fornaro M, Venerito V, Cacciapaglia F, Lopalco G. Comorbid fibromyalgia impairs the effectiveness of biologic drugs in patients with psoriatic arthritis. Rheumatol Oxf Engl. 2020;59(7):1599–606. 10.1093/rheumatology/kez505 31652315

[20] Elsawy NA, Helal AH, Abd ElHamid HA, Abdel-Fattah YH. Fibromyalgia in patients with psoriatic arthritis: impact on disease activity indices, fatigue and health‐related quality of life. Int J Rheum Dis. 2021;24(2):189–96. 33073935 10.1111/1756-185X.13987

[21] Falasinnu T, Nguyen T, Jiang TE, Chaichian Y, Rector A, Darnall BD, et al. The problem of pain in rheumatology: clinical profiles associated with concomitant diagnoses with chronic overlapping pain conditions. ACR Open Rheumatol. 2022;4(10):890–6. 35872631 10.1002/acr2.11488 PMC9555198

[22] Heidari F, Afshari M, Moosazadeh M. Prevalence of fibromyalgia in general population and patients, a systematic review and meta-analysis. Rheumatol Int. 2017;37(9):1527–39. 28447207 10.1007/s00296-017-3725-2

[23] Lubrano E, Scriffignano S, Morelli R, Perrotta FM. Assessment of widespread and extraarticular pain in psoriatic arthritis: A Case-control study. J Rheumatol. 2021;48(9):1405–9. 33452167 10.3899/jrheum.201163

[24] Taylor W, Gladman D, Helliwell P, Marchesoni A, Mease P, Mielants H, et al. Classification criteria for psoriatic arthritis: development of new criteria from a large international study. Arthritis Rheum. 2006;54(8):2665–73. 10.1002/art.21972 16871531

[25] Wolfe F, Clauw DJ, Fitzcharles MA, Goldenberg DL, Häuser W, Katz RS, et al. Fibromyalgia criteria and severity scales for clinical and epidemiological studies: a modification of the ACR preliminary diagnostic criteria for fibromyalgia. J Rheumatol. 2011;38(6):1113–22. 21285161 10.3899/jrheum.100594

[26] Nieto-Castanon A. Handbook of functional connectivity Magnetic Resonance Imaging methods in CONN [Internet]. Hilbert Press; 2020 [cited 2023 Aug 22]. Available from: https://www.hilbertpress.org/link-nieto-castanon2020

[27] Calhoun VD, Adalı T, Pekar JJ. A method for comparing group fMRI data using independent component analysis: application to visual, motor and visuomotor tasks. Magn Reson Imaging. 2004;22(9):1181–91. 15607089 10.1016/j.mri.2004.09.004

[28] Kaplan CM, Schrepf A, Ichesco E, Larkin T, Harte SE, Harris RE, et al. Association of inflammation with pronociceptive brain connections in rheumatoid arthritis patients with concomitant fibromyalgia. Arthritis Rheumatol. 2020;72(1):41–6. 31379121 10.1002/art.41069

[29] Schrepf A, Kaplan CM, Ichesco E, Larkin T, Harte SE, Harris RE, et al. A multi-modal MRI study of the central response to inflammation in rheumatoid arthritis. Nat Commun. 2018;9(1):2243. 29884867 10.1038/s41467-018-04648-0 PMC5993749

[30] Beckmann CF, DeLuca M, Devlin JT, Smith SM. Investigations into resting-state connectivity using independent component analysis. Philos Trans R Soc Lond B Biol Sci. 2005;360(1457):1001–13. 16087444 10.1098/rstb.2005.1634 PMC1854918

[31] Smith SM, Fox PT, Miller KL, Glahn DC, Fox PM, Mackay CE, et al. Correspondence of the brain’s functional architecture during activation and rest. Proc Natl Acad Sci. 2009;106(31):13040–5. 19620724 10.1073/pnas.0905267106 PMC2722273

[32] Taylor KS, Seminowicz DA, Davis KD. Two systems of resting state connectivity between the Insula and cingulate cortex. Hum Brain Mapp. 2009;30(9):2731–45. 19072897 10.1002/hbm.20705 PMC6871122

[33] Fox MD, Snyder AZ, Vincent JL, Corbetta M, Van Essen DC, Raichle ME. The human brain is intrinsically organized into dynamic, anticorrelated functional networks. Proc Natl Acad Sci U S A. 2005;102(27):9673–8. 15976020 10.1073/pnas.0504136102 PMC1157105

[34] Hemington KS, Wu Q, Kucyi A, Inman RD, Davis KD. Abnormal cross-network functional connectivity in chronic pain and its association with clinical symptoms. Brain Struct Funct. 2016;221(8):4203–19. 26669874 10.1007/s00429-015-1161-1

[35] Mosch B, Hagena V, Herpertz S, Ruttorf M, Diers M. Neural correlates of control over pain in fibromyalgia patients. NeuroImage Clin. 2023;37:103355. 36848728 10.1016/j.nicl.2023.103355 PMC9982683

[36] Mosch B, Hagena V, Herpertz S, Diers M. Brain morphometric changes in fibromyalgia and the impact of psychometric and clinical factors: a volumetric and diffusion-tensor imaging study. Arthritis Res Ther. 2023;25:81. 37208755 10.1186/s13075-023-03064-0 PMC10197341

[37] Schreiber KL, Loggia ML, Kim J, Cahalan CM, Napadow V, Edwards RR. Painful After-Sensations in fibromyalgia are linked to catastrophizing and differences in brain response in the medial Temporal lobe. J Pain. 2017;18(7):855–67. 28300650 10.1016/j.jpain.2017.02.437 PMC6102715

[38] Jensen KB, Srinivasan P, Spaeth R, Tan Y, Kosek E, Petzke F, et al. Overlapping structural and functional brain changes in patients with Long-Term exposure to fibromyalgia pain: brain changes in Long-Term fibromyalgia. Arthritis Rheum. 2013;65(12):3293–303. 23982850 10.1002/art.38170 PMC3984030

[39] Ahmed S, Plazier M, Ost J, Stassijns G, Deleye S, Ceyssens S, et al. The effect of occipital nerve field stimulation on the descending pain pathway in patients with fibromyalgia: a water PET and EEG imaging study. BMC Neurol. 2018;18(1):191. 30419855 10.1186/s12883-018-1190-5 PMC6233518

[40] Robinson CL, Phung A, Dominguez M, Remotti E, Ricciardelli R, Momah DU, et al. Pain scales: what are they and what do they mean. Curr Pain Headache Rep. 2024;28(1):11–25. 38060102 10.1007/s11916-023-01195-2

[41] Groh A, Krieger P, Mease RA, Henderson L. Acute and chronic pain processing in the thalamocortical system of humans and animal models. Neuroscience. 2018;387:58–71. 28978414 10.1016/j.neuroscience.2017.09.042

[42] Weis S, Patil KR, Hoffstaedter F, Nostro A, Yeo BTT, Eickhoff SB. Sex classification by resting state brain connectivity. Cereb Cortex. 2020;30(2):824–35. 31251328 10.1093/cercor/bhz129 PMC7444737

[43] Joel D. Beyond the binary: rethinking sex and the brain. Neurosci Biobehav Rev. 2021;122:165–75. 33440198 10.1016/j.neubiorev.2020.11.018

[44] Esteban O, Markiewicz CJ, Blair RW, Moodie CA, Isik AI, Erramuzpe A, et al. FMRIPrep: a robust preprocessing pipeline for functional MRI. Nat Methods. 2019;16(1):111–6. 30532080 10.1038/s41592-018-0235-4 PMC6319393

[45] Koch A, Stirnberg R, Estrada S, Zeng W, Lohner V, Shahid M et al. Versatile MRI acquisition and processing protocol for population-based neuroimaging. Nat Protoc. Pubblished online Deccember 13, 2024;1–23. 10.1038/s41596-024-01085-w 10.1038/s41596-024-01085-w 39672917

