# Supplementary material for: The insula represents a key neurobiological pain hub in psoriatic arthritis
Source: Arthritis Res Ther. 2025 Mar 31;27:70. doi: 10.1186/s13075-025-03526-7 (PMC11956455; doi:10.1186/s13075-025-03526-7)
Supplement: Supplementary file 1 — Supplementary Material 1 [file 13075_2025_3526_MOESM1_ESM.docx]

**Supplementary material**

(Supplementary material comprises of data (including text), tables, and figures, and should be referenced in the following format: Supplementary Data S1, Supplementary Table S1, Supplementary Figure S1.)

Figure S1: Distribution of 2011 ACR FM criteria scores in the recruited participants.

Figure S1:

The 2011 ACR FM total scores are illustrated. Each participant included in the study is represent with a histogram bar. Total FMness scores are presented in ascending order (n=46).

Table S1: Baseline clinical characteristics of participants excluded.

| **Clinical features of excluded participants**  (n=4) | |
| --- | --- |
| Age (mean ± SD) | ﻿47.4 ± 11.2 |
| Disease duration (years, mean ± SD) | 6.6 ± 6.3 |
| Sex (male/female) | 1/3 |
| BMI (mean ± SD) | ﻿29 ± 4.7 |
| FM criteria fulfilled (%) | 25% |
| FM total score (mean ± SD) | 12.6 ± 5.9 |
| Current overall body pain NRS 0-100 (mean ± SD) | 37 ± 26 |
| Number of previous DMARDs (including biologics)   - 0-1 - 2-4 - >4 | 3  1  0 |
| TJC 66 (mean ± SD) | ﻿20 ± 13 |
| SJC 68 (mean ± SD) | ﻿7.6 ± 4.7 |
| CRP (mg/dL, mean ± SD) | 1.4 ± 2.9 |
| Patient gVAS (mean ± SD) | ﻿60.6 ± 22.7 |
| DAPSA (mean ± SD) | 41 ± 20 |

BASDAI – Bath Ankylosing Spondylitis Disease Activity Index; BMI – Body Mass Index; DAPSA - Disease Activity in PSoriatic Arthritis; FM – Fibromyalgia; gVAS – global disease activity; LEI – Leed Enthesitis Index; NRS – Numeric Rating Scale; SD – Standard Deviation; SJC – Swollen Joints Count; TJC – Tender Joints Count.

Table S2: Connectivity associations with pain.

| Seed | Region | x | y | z | Cluster size | df | T Statistic | p value FDR | Effect size R^2^ |
| --- | --- | --- | --- | --- | --- | --- | --- | --- | --- |
| **Fibromyalgia** | | | | | | | | |  |
| DMN | Frontal Pole Left | -20 | 48 | 4 | 84 | 42 | -5.32 | **0.023** | **0.418** |
|  | RantIC | 32 | 16 | 6 | sphere | 42 | 2.03 | **0.048†** | **0.159** |
|  | RmidIC | 38 | 2 | 8 | sphere | 42 | 1.76 | 0.085† | - |
|  | LmidIC | -38 | 2 | 8 | sphere | 42 | 1.19 | 0.242† | - |
|  | RpIC | 39 | -15 | 8 | sphere | 42 | -0.99 | 0.326† | - |
|  | LantIC | -32 | 16 | 6 | sphere | 42 | 0.78 | 0.439† | - |
|  | LpIC | -39 | -15 | 1 | sphere | 42 | 0.63 | 0.531† | - |
| RmidIC | Parahippocampal Gyrus Left | -42 | -18 | -32 | 73 | 42 | 6.25 | **0.008** | **0.498** |
|  | Parahippocampal Gyrus Right | 20 | -14 | -36 | 74 | 42 | 5.73 | **0.008** | **0.473** |
|  | Frontal Pole Left | -32 | 64 | 6 | 119 | 42 | 4.67 | **0.001** | **0.348** |
| LmidIC | Cerebellum 8 Left | -20 | -46 | -52 | 277 | 42 | -7.81 | **<0.001** | **0.638** |
| RpIC | Thalamus Right | 14 | -6 | 14 | 150 | 42 | 5.24 | **<0.001** | **0.399** |
|  | Cerebellum 6 Right | 32 | -58 | -24 | 67 | 42 | 4.53 | **0.011** | **0.337** |
| LpIC | Parahippocampal Gyrus Right | 18 | -14 | -38 | 78 | 42 | 5.28 | **0.013** | **0.399** |
| **Current overall body pain** | | | | | | | | |  |
| RpIC | Cerebellum 7b Right | 36 | -78 | -52 | 72 | 42 | 5.82 | **0.046** | **0.471** |
| LpIC | Brainstem | -6 | -18 | -26 | 122 | 42 | 5.92 | **0.002** | **0.493** |
|  | Thalamus Left | -16 | -26 | 6 | 79 | 42 | 5.57 | **0.01** | **0.464** |
|  | Insula Right | 28 | 14 | 6 | 102 | 42 | 4.88 | **0.004** | **0.429** |
|  | Middle Temporal Gyrus Left | -52 | -62 | 12 | 66 | 42 | 4.38 | **0.017** | **0.317** |

The table displays all results of seed-to-voxel and ROI-to-ROI analyses with both nociplastic pain and current overall body pain. The seeds include the default mode network (DMN), the right (R) and left (L) anterior (ant), mid, and posterior (p) insular cortex (IC). The columns include the peak voxel MNI coordinates (x, y, z), cluster size, degrees of freedom (df), T statistic, effect size (R^2^) and p values after false discovery rate (FDR) correction for multiple comparisons of the general linear models after controlling for age and gender. ^†^Not false discovery rate (FDR) multiple comparisons corrected.
